# Experiments of Main Parameters Affecting the Erosive Behavior of Self-Excited Oscillating Abrasive Water Jets: Length of Self-Oscillation Chamber, Jet Pressure, Abrasive Fluid Velocity, and Abrasive Grain Size

**DOI:** 10.3390/ma17143449

**Published:** 2024-07-12

**Authors:** Baochun Tao, Chengqi Zhang, Qianfa Deng, Qiming Wang, Hong Zhang, Lizhi Sun

**Affiliations:** 1 Special Equipment Institute, Hangzhou Vocational & Technical College, Hangzhou 310018, China; taobc@sina.com; 2 Ultra-Precision Machining Center, Zhejiang University of Technology, Hangzhou 310023, China; 3 Key Laboratory of Special Purpose Equipment and Advanced Manufacturing Technology, Zhejiang University of Technology, Ministry of Education, Hangzhou 310023, China; 4 Department of Civil & Environmental Engineering, University of California, Irvine, CA 92697, USA

**Keywords:** abrasive water jet, fluid self-excited oscillation, erosion, SiC material removal

## Abstract

To enhance the erosion efficiency in traditional abrasive water jet processing, an abrasive water jet processing method based on self-excited fluid oscillation is proposed. Traditional abrasive water jet methods suffer from reduced jet kinetic energy due to the presence of a stagnation layer, which hinders efficient material removal. By integrating a self-oscillation chamber into the conventional abrasive water jet nozzle, the continuous jet is transformed into a pulsed jet, thereby increasing the jet velocity and enhancing the kinetic energy of the process. This modification aims to improve material removal efficiency. Using Ansys Fluent, we simulated the material removal efficiency on workpiece surfaces with varying lengths of self-oscillation chambers. The simulation results reveal that the optimal length of the self-oscillation chamber for maximum erosion is 4 mm. SiC materials were used to evaluate the impact of self-oscillation chamber length (L), jet pressure (P), abrasive flow rate (M), and abrasive grain size (D) on erosion. Experimental results show that the self-oscillation chamber increases erosion depth by 33 μm. The maximum erosion depths recorded were 167 μm when L = 4 mm, 223 μm when P = 16 MPa, 193 μm when M = 80 g/min, and 268 μm when D = 2000 μm. Overall, the self-excited oscillation effect enhances the erosion efficiency of the waterjet by 14%. This study further elucidates the factors influencing erosion behaviors in oscillating abrasive water jet processing.

## 1. Introduction

Silicon carbide (SiC) is the most widely used high-performance material in semiconductor, optics, and industrial manufacturing [1,2]. It is characterized by high hardness, high melting point, high chemical stability, and excellent wear resistance [3]. However, due to its extremely high hardness and brittleness, traditional machining methods face significant challenges in SiC material machining [4]. These challenges include susceptibility to cracking and low machining efficiency [5]. In order to overcome these difficulties, abrasive water jet processing has been widely studied and applied as a new and efficient processing method. Abrasive water jet technology employs high-pressure water jets to propel abrasive particles, which then cut and remove materials through the kinetic energy of abrasive. This enables the efficient processing of hard–brittle materials such as SiC [6,7,8,9]. In contrast to traditional machining techniques, the abrasive water jet machining process is devoid of thermal influence, thus preventing the formation of material deformations and cracks due to frictional heat. This ensures the precision and surface quality of machined parts [10].

To gain a deeper understanding of the processing capabilities of abrasive water jet technology, researchers have conducted extensive studies into the technology’s performance and underlying mechanisms. The objective of these studies has been to enhance the technology’s efficiency and precision control. Tie et al. [11] used the method of rotating abrasive water jet nozzles in the CCOS (computer-controlled optical surface forming) machining of optical components to optimize the removal function of conventional abrasive water jets and obtained a Gaussian form of the removal function, which was experimentally verified to be stable in terms of material removal capability. This method significantly improved the forming capability of conventional abrasive water jets and the fabrication capability of optical components, although its application is mainly limited to optical component fabrication. Wang et al. [12] applied the micro-abrasive slurry jet (MASJ) polishing technology to the post-treatment of PVD-coated tools, and by optimizing the process parameters such as jet pressure, abrasive particle size, angle of incidence, and abrasive mass concentration, the lowest roughness and the highest removal of the coating were obtained. This contributed to the application of abrasive water jets to thin surfaces for micro-determined removal, providing high precision and low surface roughness, although it is mainly applicable to thin surfaces.

In addition to the optimization of the abrasive water jet processing process, the researchers also proposed several alternative technologies, including laser-assisted abrasive water jet technology [13], ultrasonic vibration-assisted abrasive water jet technology [14], electrochemical-assisted abrasive water jet technology [15], cavitation-assisted abrasive water jet technology [16], and other composite abrasive water jet processing technology [17]. These technologies are designed to address the limitations of traditional abrasive water jet technology, including low efficiency and instability, but often at the cost of increased complexity and potentially higher expenses.

To enhance the efficiency and quality of machining, researchers have recently turned their attention to self-excited oscillating abrasive water jet technology. This technology exploits the self-excited oscillation phenomenon, which arises from the interaction between abrasive particles and water jets during processing. This phenomenon stabilizes the processing and enables higher efficiency and superior quality. Consequently, the introduction of self-excited oscillating abrasive waterjet technology into the field of SiC material processing is anticipated to facilitate further expansion of its application scope and enhance processing efficiency, thereby aligning with the manufacturing industry’s demand for high-performance material processing. In a series of experiments on sandstone, Zhang et al. [18] employed a pulsed abrasive supercritical carbon dioxide jet to investigate the effects of oscillating cavity length ratio and jet pressure on the erosion properties of the rock. Their findings indicated that the depth of erosion was optimal when the ratio of cavity length to the diameter of the upper nozzle was 4 and that the depth and width of erosion were linearly correlated with the jet pressure. This provided clear optimization parameters for maximum erosion, although it was specific to sandstone and supercritical carbon dioxide jets. Xiang et al. [19] employed FLUENT software to investigate the impact of the nozzle outlet on the oscillation characteristics of the jet within the oscillating cavity. Their findings indicated that the length and thickness of the outlet projection of the upper nozzle exerted a significant influence on the self-oscillating oscillatory characteristics. This offered a detailed understanding of the nozzle’s impact on oscillation, although it was limited to simulation results. Liu et al. [20] studied the effect of the nozzle pressure ratio on the frequency of the free-flow field. The results indicated that the frequency exhibited a fluctuating trend as the pressure ratio of the nozzle increased. This provided insight into pressure ratio effects on frequency, though it focused mainly on the pressure ratio without extensive practical application.

This paper introduces the fundamental principles of self-excited oscillating pulsed abrasive water jet technology and the mechanisms underlying erosion behavior during material processing. It employs numerical simulation to assess the material erosion of three distinct self-oscillation chamber lengths under varying parameters, integrating Ansys Fluent (2021 R1) software. Finally, it conducts material removal experiments under the optimal self-oscillation chamber lengths and analyzes the influence of the relevant process parameters. The parameters were varied to examine their influence on the erosion pattern and the erosion depth of the SiC processed by abrasive water jets with self-excited oscillating fluid characteristics. Results were compared with those of the SiC polished by conventional abrasive water jets. It found that self-excited oscillating pulsed jets greatly enhance the eroding capacity of abrasive water jets in processing SiC materials.

## 2. Processing Principle

**Figure 1:**
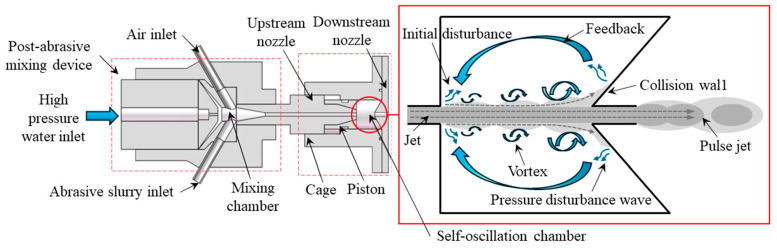
Schematic of self-excited oscillating abrasive water jet.

A diagram of a self-excited oscillation abrasive water jet processing principle is shown in Figure 1 [21], which includes the high-pressure water, formed by the piston pump pressurization, flowing from the inlet into the mixing chamber. The abrasive slurry is fully mixed into a polishing solution in a mixing chamber that has two inlets, one connected to the atmosphere, and the other used as the abrasive slurry inlet to be able to realize the arbitrary adjustment of the concentration of the abrasive material and precise control [22]. The polishing solution moves from the upper nozzle into the self-oscillation chamber, where it collides with the unstable shear layer inside the self-oscillation chamber and forms the initial perturbation. Then, further selective amplification is performed and the perturbation continues to move downstream the collision wall, resulting in a feedback pressure wave. At a certain speed, the perturbation moves upstream to the feedback, so that the upstream inlet and a new perturbation are formed. This process is repeated when the downstream collision wall and the upstream inlet are generated by the same perturbation phase, which will then be able to form a cyclic continuous feedback. At this time, the upper nozzle inlet’s continuous jet converted to a self-excited oscillating pulsed characteristic of the jet at the lower nozzle outlet, which can cause extreme damage to the stagnant layer [23,24], and thus increase the processing capacity of the jet.

## 3. Simulation Model

### 3.1. Fluid Control Modeling

In this experiment, the simulation analyzes the flow field inside the self-oscillation chamber and the flow field of the jets eroding the surface of the workpiece. The external flow field is enabled by the mixture multiphase flow model, which sets the water as the first phase and the abrasive material as the second phase because there are more eddies inside the self-oscillation chamber. The simulation presents in detail the erosion situation of the near-wall surface, so the turbulence model adopts the Realizable k-ε model, which has high curvature and strong vortex flow [25], and is suitable to be used as the prototype jet. Its calculations are more accurate in simulating the near-wall flow, and the back-pressure gradients are adapted well in accordance with the research of this topic.

The medium in the simulation is an incompressible fluid with the following continuity equation:

(1) $$\begin{array}{c} \frac{d\rho }{dt}+\rho \frac{\partial u_{i}}{\partial x_{i}},\;i=1,\;2,\;3\; \end{array}$$

And for an incompressible fluid like water, $\frac{d\rho }{dt}=0$, so its continuity equation can be rewritten as

(2) $$\begin{array}{c} \frac{\partial u_{i}}{\partial x_{i}}=0,\;i=1,\;2,\;3\; \end{array}$$

whose momentum equation can be described as

(3) $$\begin{array}{c} \frac{\partial {\bar{v}}_{i}}{\partial t}+\frac{\partial {\bar{v}}_{i}{\bar{v}}_{j}}{\partial x_{j}}=-\frac{1}{\rho }\frac{\partial \bar{p}}{\partial x_{i}}+\sigma \frac{\partial^{2}{\bar{v}}_{i}}{\partial x_{i}\partial x_{j}}-\frac{\partial {\bar{\tau }}_{ij}}{\partial x_{j}}\; \end{array}$$

### 3.2. Erosion Model

Ansys Fluent gives a generalized model for calculating the erosion rate [26]:

(4) $$\begin{array}{c} R_{erosion\;}=\sum_{p=1}^{N_{particles}}\;\frac{m_{a}C\left(d_{a}\right)f\left(\theta \right)v^{b\left(v\right)}}{S_{face}}\; \end{array}$$

### 3.3. Wall Bounce Function

In the DPM erosion simulation process, the particles will rebound irregularly after impacting the workpiece, the rebound velocity and angle will be changed, and the energy loss will be large. In this paper, the velocity recovery coefficient model proposed by Grant G and Tabakoff [27] is used, which is formulated as

(5) $$\begin{array}{c} \left\{\begin{array}{c} E_{n}=0.993-1.76\theta +1.54\theta^{2}-0.49\theta^{3}\;\\ E_{t}=0.988-1.66\theta +2.11\theta^{2}-0.67\theta^{3} \end{array}\right.\; \end{array}$$

In this equation, $E_{n}$ is the normal bounce factor and $E_{t}$ is the tangential bounce factor.

The specific rebound coefficient is determined by the cavity wall material (304L stainless steel) and the type of abrasive grain (SiC), as shown in Table 1 below.

### 3.4. Boundary Conditions

**Figure 2:**
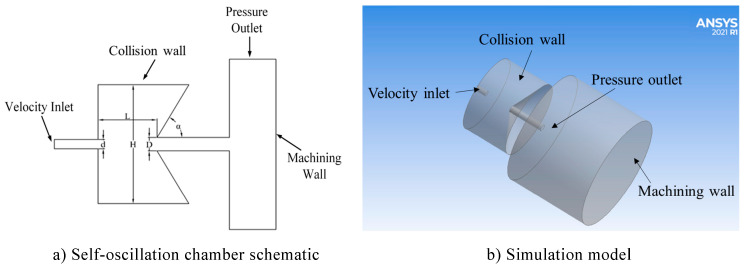
Schematic of self-oscillation chamber and boundary conditions. (**a**) Self-oscillation chamber schematic. (**b**) Simulation model.

**Figure 3:**
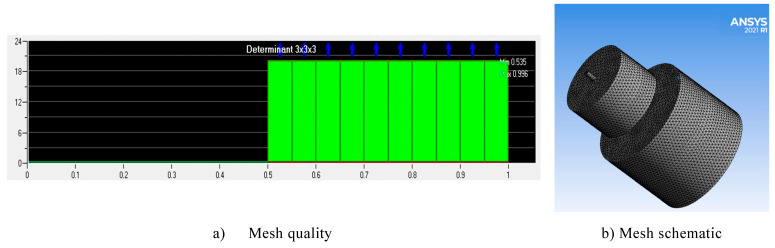
Mesh inspection and schematic diagram. (**a**) Mesh quality. (**b**) Mesh schematic.

This simulation uses ANSYS ICEM to mesh division; Figure 2 shows the structure of a self-oscillation chamber, while the structural parameters are shown in Table 2. The O-type method is used to divide and enable the structural mesh, taking into account the near-wall effect. The mesh close to the self-oscillation chamber and the machined surface near the mesh are encrypted. The overall quality of the divided mesh model is above 0.5, as shown in Figure 3. After the mesh division is completed, the Ansys Fluent software is enabled to carry out the processing flow field simulation, and structure parameters are set as shown in Table 3 below.

The abrasive grains are driven by the fluid medium during movement. To simulate this, the continuous phase calculation is initiated simultaneously with the DPM (discrete phase model). The erosion option is enabled to account for material wear. For pressure–velocity coupling, the SIMPLE algorithm is employed. The pressure discretization uses the PRESTO format, while all other parameters are set to second-order accuracy. In this simulation, both the fluid and abrasive phases use a velocity inlet boundary condition to ensure consistency. The inlet velocity of the abrasive grains matches that of the fluid. The inlet pressure is set to one atmosphere, and the outlet boundary condition is specified as a pressure outlet at one atmosphere. All walls are treated as fixed no-slip boundary conditions. The collision behavior of abrasive particles with the processing wall is set to reflect for accurate erosion calculations. Once the calculations are completed, the results are imported into CFD-POST for post-processing analysis.

The abrasive grain is driven by the fluid medium that moves. Therefore, to start the discrete phase of DPM (deformable parts model) at the same time as the continuous phase calculation, the following steps are performed: turn on the erosion option, perform the pressure–velocity coupling using SIMPLE, and conduct the pressure discrete using PRESTO Discrete format. The other parameters are all of second order. In the same fluid simulation, the abrasive phase also uses the velocity inlet as the inlet boundary, it’s velocity is consistent with the fluid velocity; the inlet pressure boundary is one atmosphere and the same with the outlet boundary; all walls are fixed no-slip boundary conditions; the collision form of abrasive particles on the processing wall is set to REFLECT. Once the calculations are completed, the results are imported into CFD-POST for post-processing analysis.

## 4. Simulation of Erosion Results

### 4.1. Particle Size

**Figure 4:**
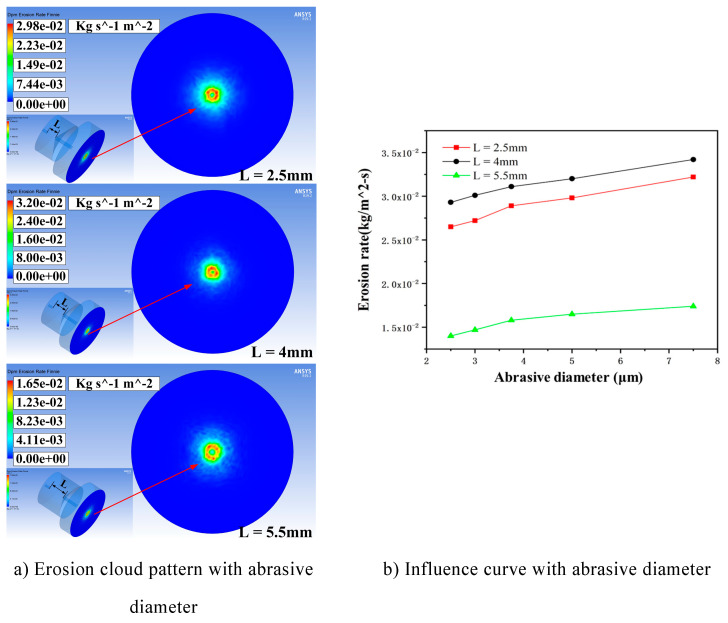
Erosion distribution cloud and influence curve under different cavity lengths affected by particle size. (**a**) Erosion cloud pattern with abrasive diameter. (**b**) Influence curve with abrasive diameter.

In the case of when the entrance speed is 135 m/s, the particle mass flow rate is 0.00067 kg/s, and the particle size is in the order of 2.5 μm, 3 μm, 3.75 μm, 5 μm, and 7.5 μm, simulations are carried out for three chamber lengths of L = 2.5 mm, 4 mm, and 5.5 mm respectively To study the effect of particle size on the erosion results of the processed wall, the simulation is analyzed when the particle size is 5 μm. The erosion distribution is shown in Figure 4a, and the influence curve of the maximum erosion rate is shown in Figure 4b.

From Figure 4, it can be seen that under the same conditions, when the cavity length is 4 mm, the maximum erosion rate is greater than the other two cavity lengths, and the maximum can be up to 0.032 kg/(m^2^s). Combined with Equation (4), the data indicate that there is a proportional relationship between the erosion rate and the particle impact velocity, i.e., the best acceleration effect on the particles of the cavity length corresponds to the higher erosion rate. And no matter the cavity length, the maximum erosion rate exhibits a linear increasing trend in response to changes in particle size, which can be interpreted as when the particle diameter of the particles is smaller—as the particle size increases, the mass also increases; the particles carry a high kinetic energy, so the erosion rate is large.

### 4.2. Particle Mass Flow Rate

**Figure 5:**
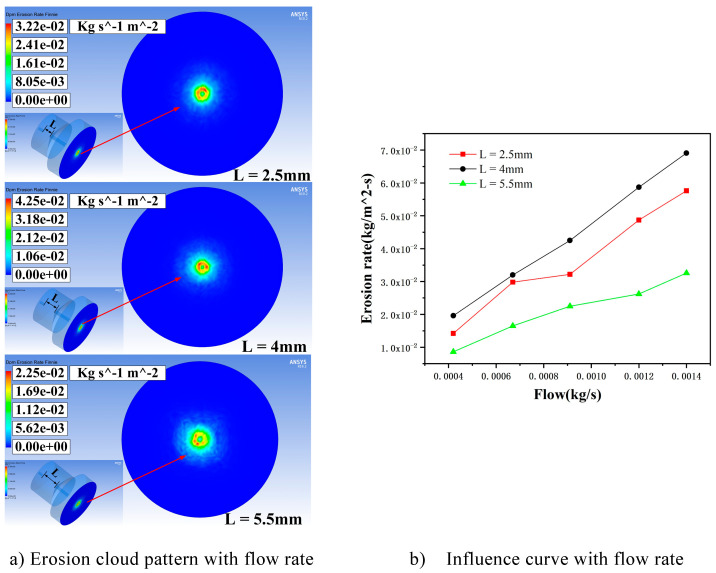
Erosion distribution cloud and influence curve under different cavity lengths affected by flow rate. (**a**) Erosion cloud pattern with flow rate. (**b**) Influence curve with flow rate.

In the case of when the inlet velocity is 135 m/s, the particle size is 5 μm, and the particle mass flow rate is 0.00042 kg/s, 0.00067 kg/s, 0.00091 kg/s, 0.0012 kg/s, and 0.0014 kg/s. Simulations were carried out for three cavity lengths of L = 2.5 mm, 4 mm, and 5.5 mm, respectively, to study the effect of particle mass flow rate on the erosion results of the machined wall, and the simulations were taken to be analyzed when the flow rate was 0.00091 kg/s. The erosion distribution is shown in Figure 5a, and the curve of the effect of the maximum erosion rate is shown in Figure 5b.

In summary, when the cavity length is short, the vortex ring cannot be effectively amplified in the cavity and fed back into the jet beam, resulting in a weak acceleration of the jet beam. When the cavity length is too long, the vortex ring will develop excessively, and the excessive perturbation generated causes the jet to hit the collision wall several times, resulting in kinetic energy loss. From Figure 5, when the cavity length is 4mm, the maximum erosion rate achieves the maximum value, up to 0.0691 kg/(m^2^s). The maximum erosion rate and particle mass flow rate are proportional to the case; this is because when the inlet velocity and particle size remain unchanged, as particle mass flow rate increases, the total number of particles participating in the erosion of the wall also increases linearly. The wall by the frequency of impact is also greater, so the erosion rate is linearly increased.

### 4.3. Inlet Velocity

**Figure 6:**
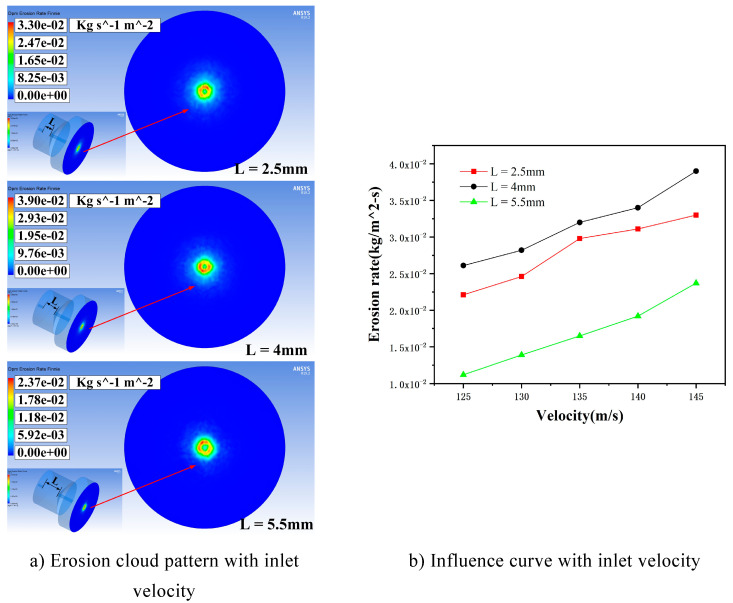
Erosion distribution cloud and influence curve under different cavity lengths affected by inlet velocity. (**a**) Erosion cloud pattern with inlet velocity. (**b**) Influence curve with inlet velocity.

For a particle size of 5 μm, the particle mass flow rate is 0.00067 kg/s and the inlet velocity is 125 m/s, 130 m/s, 135 m/s, 140 m/s, and 145 m/s. Simulations were carried out for three cavity lengths of L = 2.5 mm, 4 mm, and 5.5 mm, respectively, to study the inlet velocity on the erosion results of the machined wall, and the simulations were taken to be analyzed when the entrance speed was 145 m/s. The erosion distribution is shown in Figure 6a, and the curve of the effect of the maximum erosion rate is shown in Figure 6b.

Figure 6 illustrates that, in accordance with the particle mass flow rate and particle size, while maintaining consistent simulation parameters, the maximum erosion rate at a cavity length of 4mm is greater than the other two cavity lengths, reaching a maximum of 0.039 kg/(m^2^s). The erosion rate is proportional to the inlet velocity because, as the fluid velocity increases, the accelerated abrasive particles carried by it also gain greater kinetic energy, enhancing its impact ability on the wall. At the same time, an increase in flow velocity will lead to an increase in turbulent kinetic energy; this makes more particles collide with the surface, which worsens erosion.

## 5. Experimental Conditions and Methodology

### 5.1. Experimental Setup

**Figure 7:**
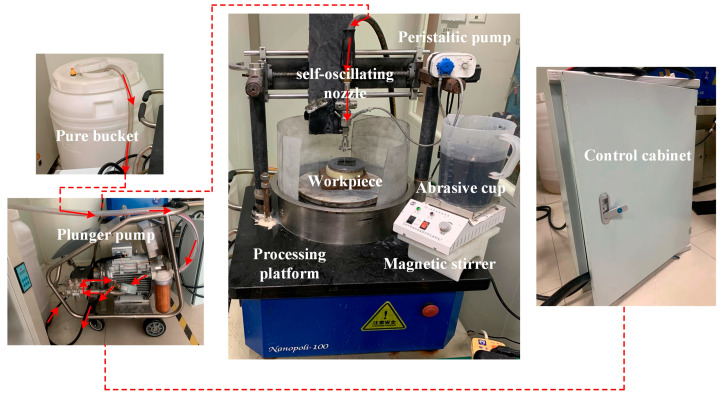
Self-excited oscillating abrasive water jet processing platform and direction of flow.

**Figure 8:**
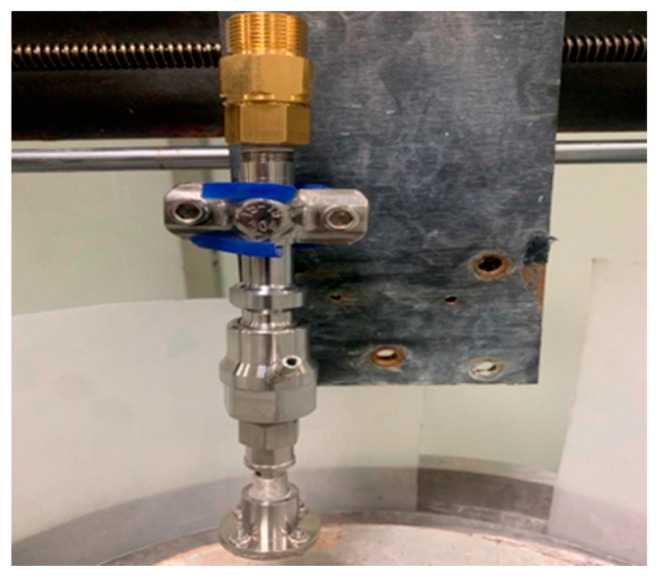
Self-oscillation chamber.

Figure 7 shows the experimental platform for self-excited oscillating abrasive water jets. This experimental platform mainly consists of a high-pressure pump, an abrasive pump, a nozzle, a bucket, a frequency converter, etc. The plunger pump affects how well the jet works. A high-pressure pump with a pressure of up to 25 MPa was chosen. The pure water was pressurized to a set pressure through the high-pressure pump and then stabilized through an accumulator. It was then put into the self-oscillation chamber. At the same time, the abrasive pump extracts the abrasive slurry into the mixing chamber, mixing it with the pure water. A high-pressure abrasive water jet is formed and then enters the self-oscillation chamber. The water-jet-processing equipment and self-oscillation chamber material is CNC-fabricated using 304L stainless steel and designed to last 50 h with less than a five percent change in outlet diameter due to wear. The whole self-excited oscillation nozzle is fixed on the machining spindle, which can adjust the distance and angle. The length of the self-excited oscillation cavity can be changed to make different types of abrasive water jets with different speeds, as shown in Figure 8.

### 5.2. Experimental Materials

**Figure 9:**
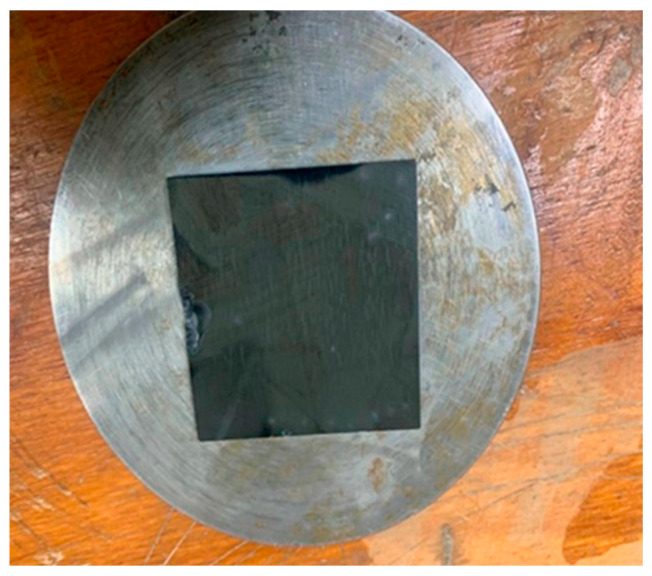
Silicon carbide wafer.

The materials of this experimental workpiece are 20 mm × 20 mm × 2.2 mm silicon carbide substrates as shown in Figure 9, the abrasive choice of SiC particles, polishing solution from deionized water, SiC abrasive particles, and a certain proportion of the dispersant configuration.

### 5.3. Experiment

In the pulsed abrasive water jet erosion process, the experiment is mainly about the pressure, grain size, and flow rate of the jet. Each variable is tested at five levels. The presence or absence of the self-excited oscillating cavity structure is also tested. We got 30 sets of material removal data, shown in Table 4.

The workpiece was fixed on the platform and kept perpendicular to the jet. The target distance was 10 mm, the abrasive concentration was 5%, and the silicon carbide sheet was spot-etched. Each experiment took about 10 min. At the end of the experiment, a microscope (VHX-7000, KEYENCE, Shanghai, China) was used to observe and measure the erosion on the workpiece.

## 6. Results and Discussion

### 6.1. Self-Oscillation Chamber Length

**Figure 10:**
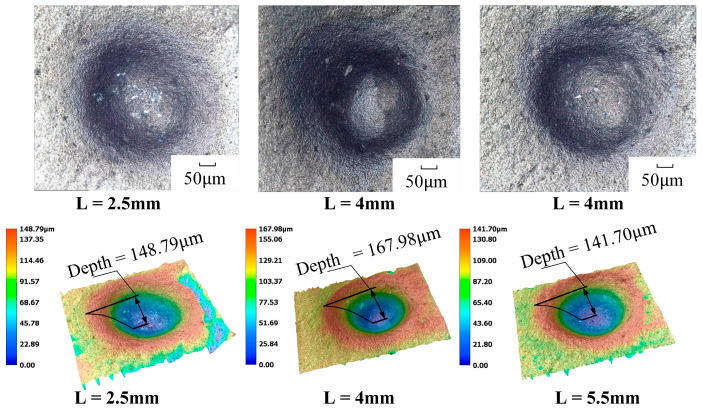
Erosion morphology and depth comparison of different cavity lengths.

Before the start of the experiment, a self-exciting cavity length erosion verification experiment was conducted to demonstrate the superiority of the erosion effect at L = 4 mm (Table 4), with the rest of the parameters remaining unchanged, and the experiment was carried out at a jet pressure of 12 MPa, an abrasive grit of 1500#, and an abrasive flow rate of 50 mL/min. The workpiece material was subjected to single-point erosion at three different locations, with the application of three distinct cavity lengths (L = 2.5 mm, 4 mm, and 5.5 mm) for a period of ten minutes. This process yielded three erosion stationary points.. The erosion results are shown in Figure 10.

From Figure 10, under the same experimental conditions, the three cavity lengths are able to erode a more obvious pit. The erosion depth in the figure represents the straight-line distance from the lowest point to the highest point in the pit, and the erosion depth is the largest when L = 4 mm, which can reach 167.98 μm, which is consistent with the results obtained from the simulation with a maximum removal rate of L = 4 mm. This proves that the strongest removal ability of the jet is achieved when the cavity length is 4 mm.

### 6.2. Jet Pressure

**Figure 11:**
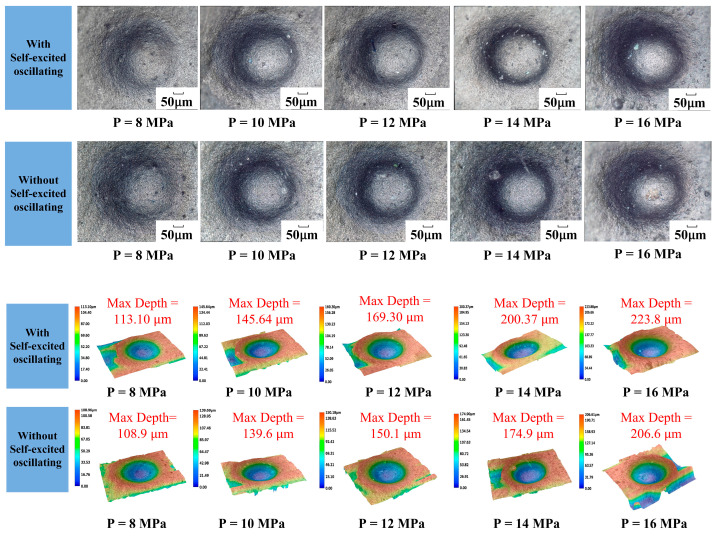
Erosion pattern and depth of cavities under different jet pressures.

The one-factor experiment of jet pressure was carried out by taking the cavity length of 4 mm; the abrasive grain size was 1000#, the abrasive flow rate was 50 mL/min, and the rest of the parameters are shown in Table 4. Figure 11 shows the comparison of erosion morphology and depth with and without a self-excited oscillating cavity structure. The experiment on jet pressure was conducted with a 4 mm chamber, 1000# abrasive, 50 mL/min flow, and the other parameters listed in Table 4. Figure 11 shows erosion patterns and depths with and without the oscillating cavity structure.

**Figure 12:**
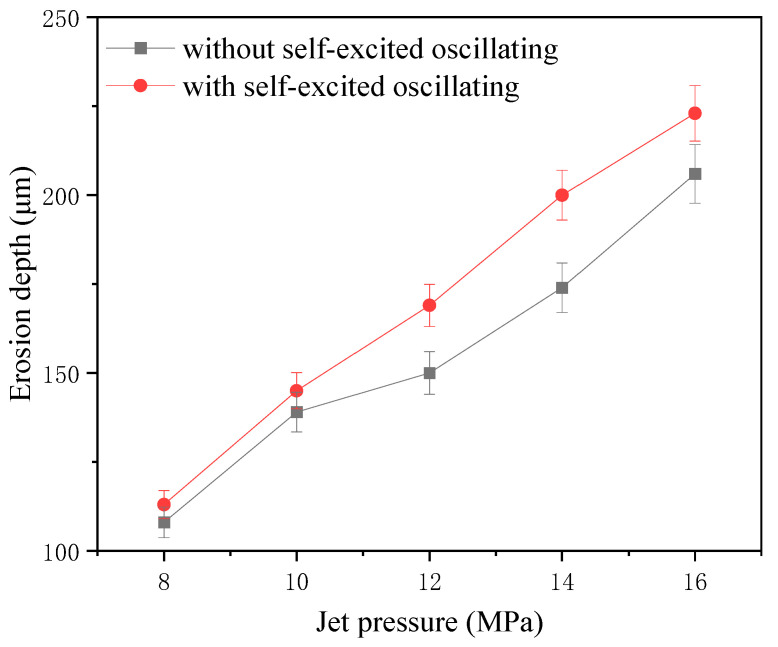
Erosion depth curve under different jet pressures.

The impact curve is shown in Figure 12. The results of processing using the self-excited oscillating water jet indicate that the impact force at the depth of erosion is greater than that achieved through conventional abrasive water jet polishing methods. The difference between the two depths can reach up to 26 μm, and the depth of the erosion increases when the jet pressure increases. The maximum can be up to 223 μm, which indicates that the pressure of the water jet in the mixing chamber can be converted to the kinetic energy of the abrasive particles. This can be explained by the fact that the pressure energy of the water jet in the mixing chamber is converted into the kinetic energy of the abrasive particles, and the increase in jet pressure directly leads to the increase in the kinetic energy of the abrasive particles, which improves the erosion performance of the individual abrasive particles, i.e., the vertical impact on the surface of the material and the ability to remove the material by shear. This in turn increases the depth of erosion.

### 6.3. Abrasive Flow

**Figure 13:**
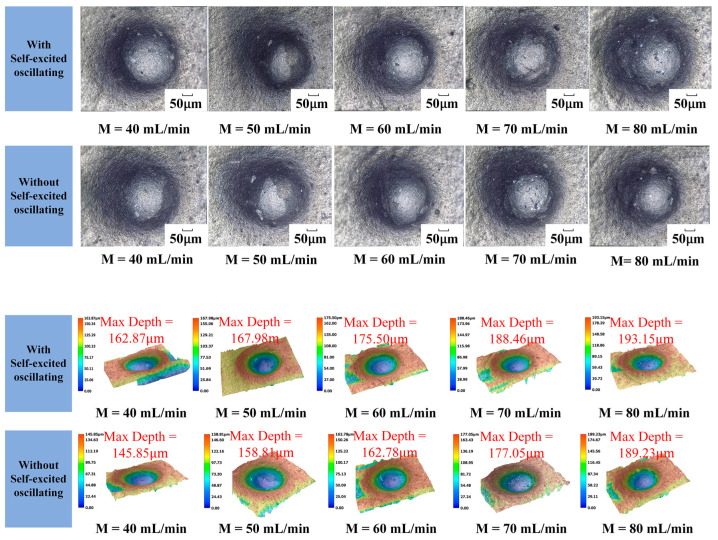
Erosion pattern and depth under different flow rates.

The one-factor experiment of abrasive flow rate was carried out by taking a cavity length of 4 mm, an abrasive grain size of 1000#, a jet pressure of 12 MPa, and the rest of the parameters shown in Table 4. Figure 13 shows the erosion pattern and depth with and without a self-oscillation chamber.

**Figure 14:**
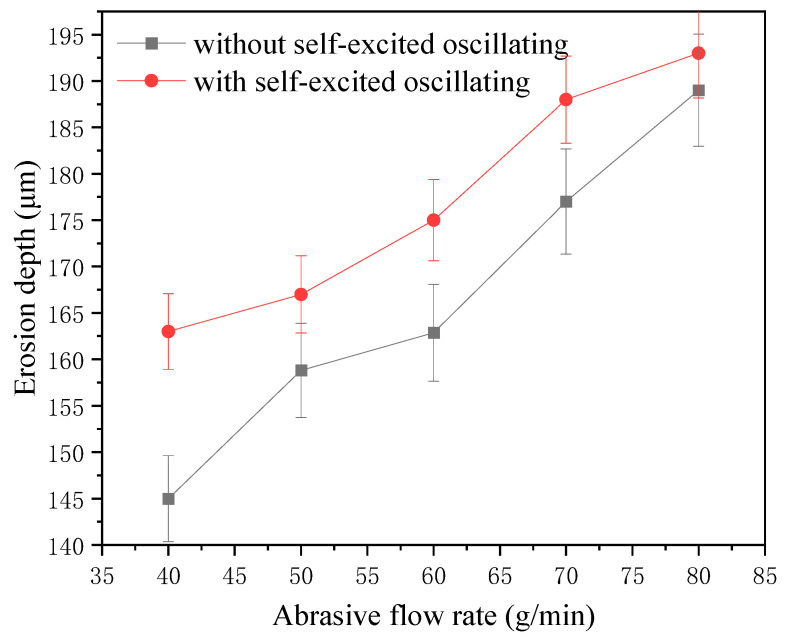
Erosion depth curve under different flow rates.

The influence curve is shown in Figure 14. It can be seen that the self-excited oscillating method has a greater effect on the depth of erosion than the conventional abrasive water jet polishing method. The difference between the two depths can reach up to 18 μm, the erosion depth increased linearly with abrasive flow in both cases. In the jet system with a self-oscillation chamber, the maximum can be up to 193 μm; this is because the abrasive flow rate directly determine the number of abrasive particles participating in erosion per unit time. This also promotes the interaction between the abrasive particles, thus changing the total kinetic energy of the particles. When the jet pressure and the particle size are both of a certain amount, the abrasive flow increases. On the one hand, this can improve the total kinetic energy of the particles, but on the other hand, it will also increase the possibility of mutual collision between the particles. These changes will result in energy loss, leading to a decrease in the corrosion performance of abrasive particles.

### 6.4. Abrasive Grain Size

**Figure 15:**
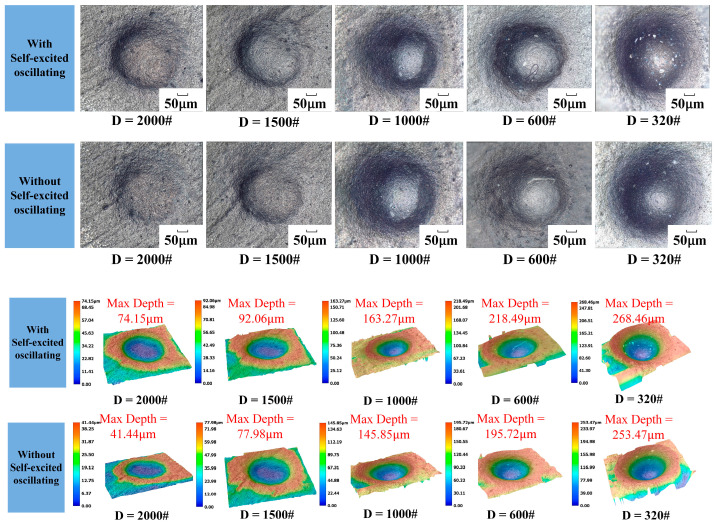
Erosion pattern and depth under different particle sizes.

The one-factor experiment of abrasive grain size was carried out by taking a cavity length of 4 mm, an abrasive flow rate of 50 mL/min, a jet pressure of 12 MPa, and the rest of the parameters shown in Table 4. Figure 15 shows the erosion pattern and depth maps with and without the self-oscillation chamber.

**Figure 16:**
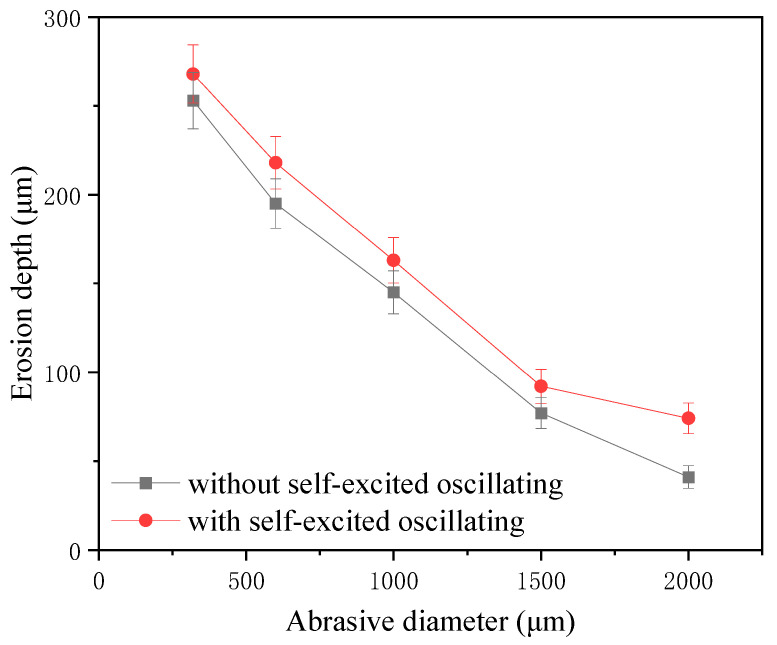
Erosion depth curve under different particle sizes.

The impact curve is shown in Figure 16. It can be seen in the structure with a self-excited oscillating cavity that the impact under the erosion depth is greater than when with the traditional abrasive water jet polishing. The difference between the two depths can reach up to 33 μm, illustrating the depth of erosion in both cases, and the abrasive flow rate is a linear growth relationship with a self-excited oscillating cavity that can be as large as 268 μm. This is because not only does the level of the kinetic energy of the individual particles directly affect the impact of the particles on the surface of the material, but it also leads to greater contact with the area, which results in a higher removal of the amount of materials to lead to a greater removal of the depth.

## 7. Conclusions

In this paper, experiments involving the processing of self-excited oscillating pulsed abrasive water jet using SiC were carried out to study the removal characteristics of the material. Firstly, a simulation was conducted to determine the optimal cavity length for erosion and the influence of process parameters on the surface erosion rate of the workpiece. Subsequently, experiments were carried out on self-excited oscillating abrasive water jet processing SiC with/without a self-oscillation chamber, with the aim of verifying the influence of changes in process parameters on the morphology of the workpiece micro-crater and the depth of erosion. The following main conclusions are drawn:

(1) The results of the self-excited oscillating abrasive water jet erosion simulation indicated that when the cavity length was 4mm, the material erosion rate was the highest. Furthermore, the erosion rate and the inlet velocity, abrasive grain size, and abrasive flow rate exhibited a linear growth relationship. Inlet velocity and abrasive flow rate had the greatest influence on the erosion effect, followed by abrasive grain size.

(2) A comparison of the erosion depth of micro-craters on the SiC surface after machining revealed that the self-excited oscillating abrasive waterjet can cause a greater removal depth than the normal abrasive waterjet. The depth was found to have been expanded by 33μm, with a maximum depth increase of 14%.

(3) The impact of various processing parameters on the depth of erosion was examined. It was determined that an increase in jet pressure, abrasive grit size, and abrasive flow rate resulted in a corresponding increase in erosion depth. A broad adjustable range of abrasive grit size exhibited the most pronounced effect on erosion depth. A maximum erosion depth of 268 μm was attained when utilizing a self-excited oscillating chamber.

## 8. Outlook

(1) More structural or experimental parameters of the cavity can be simulated, including the effects of the angle of the collision wall and the diameter of the entrance/exit of the self-excited cavity on the self-excited pulsed characteristics of the fluid, so as to guide the experiments more accurately.

(2) In the future, ultrasonic vibration, magnetic field, and other auxiliary field processing means can be combined to further enhance the fluid pulsed characteristics to improve the processing quality and efficiency.

(3) This paper only focuses on the single-point erosion behavior of silicon carbide materials, and in the future, more hard and brittle materials or complex curved surfaces and optical glass surfaces can be eroded as a whole.

## Figures and Tables

**Table 1:** Wall rebound function table.

		Coefficients		
$E_{n}$	0.993	1.76	1.54	−0.49
$E_{t}$	0.998	−1.66	2.11	−0.67

**Table 2:** Main structure parameters of self-excited oscillation.

D/d	1.32
H/d	5.92
L (mm)	2.5, 4, 5.5
α (°)	60
d (mm)	0.76

**Table 3:** The main settings of simulation.

Parameters	Values
Water density/$\left(kg\cdot m^{-3}\right)$	998
Inlet velocity V/$\left(m\cdot s^{-1}\right)$ Particle density/$\left(kg\cdot m^{-3}\right)$ Particle mass flow rate/$\left(kg\cdot s^{-1}\right)$	125~145 3515 0.00042~0.0012
Particle size/μm Gravitational acceleration/$\left(m\cdot s^{-2}\right)$	2.5~6.5 9.8
Hydraulic diameter/mm	55

**Table 4:** Experimental parameters.

Jet Pressure P/MPa	Abrasive Particle Size D(#)	Target Distance H/mm	Erosion Angle α/(°)	Abrasive Concentration W/(wt%)	Abrasive Flow M/(mL/min)
8, 10, 12, 14, 16	320~2000 SiC	10	90°	5	40, 50, 60, 70, 80

## Data Availability

The raw data supporting the conclusions of this article will be made available by the authors on request.

## List of Symbols

ρ	Density
$v_{i}$	Instantaneous velocity
$x_{i}$	Three-dimensional coordinate direction
$\tau_{ij}$	Modified subgrid scale tensor
$R_{erosion}$	Erosion rate
$N_{particles}$	Number of particles
$m_{a}$	Particle mass flow
$C\left(d_{a}\right)$	Particle size function
$f\left(\theta \right)$	Impact angle function
$S_{face}$	Processing wall area
D/d	Lower nozzle diameter/upper nozzle diameter
H/d	Chamber diameter/upper nozzle diameter
L	Length of cavity
α	The angle of the collision wall
V	Inlet velocity
P	Jet pressure
D	Abrasive particle size
H	Target distance
W	Abrasive concentration
M	Abrasive flow
